# Epidemiology of Parkinson's Disease in Germany Between 2016 and 2021 Based on Statutory Health Insurance Claims Data

**DOI:** 10.1002/brb3.71027

**Published:** 2025-11-05

**Authors:** Dirk Woitalla, Gisa Ellrichmann, Stefan Jun Groiss, Stefan Walzer, Lutz‐Michael Vollmer, Signe Thiesen‐Nash, Sebastian Krenberger, Julia Borchert, Glynn Harrison‐Jones

**Affiliations:** ^1^ Department of Neurology St. Josef‐Krankenhaus Kupferdreh Essen Germany; ^2^ Faculty of Health/School of Medicine, Chair of Neurology II Witten/Herdecke University Witten Germany; ^3^ Department of Neurology Klinikum Dortmund gGmbH Dortmund Germany; ^4^ Neuro‐Centrum Düsseldorf Düsseldorf Germany; ^5^ MArS Market Access & Pricing Strategy GmbH Weil am Rhein Germany; ^6^ State University Baden‐Wuerttemberg Loerrach Germany; ^7^ University of Tübingen, Geoinformatics Tübingen Germany; ^8^ University of Applied Sciences Rottenburg/Neckar Rottenburg Germany; ^9^ BIAL Deutschland GmbH Mörfelden‐Walldorf Germany; ^10^ WIG2 GmbH Leipzig Germany; ^11^ BIAL Pharma UK Ltd. Windsor UK

**Keywords:** epidemiology of Parkinson's disease by gender, incidence of Parkinson's disease, Parkinson's disease with dementia, prevalence of Parkinson's disease

## Abstract

**Introduction:**

Parkinson's disease (PD) is a fast‐growing neurodegenerative disease causing a high burden on national health systems. There is a lack of contemporary, reliable epidemiological data for PD in Germany. The aim of this study was to estimate the incidence and prevalence of PD in Germany for the years 2016–2021.

**Methods:**

This is a secondary data analysis of anonymized health claims data from a subset of German statutory health insurances (SHIs) for the years 2016–2021. Data were then extrapolated to represent the full SHI population.

**Results:**

From 2016 to 2021, PD prevalence in the study population increased from 494 to 511 patients per 100,000 (*p* = 0.0067). Incident patients showed no change overall but notably decreased from 2019 to 2020. In contrast, PD prevalence in the extrapolated cohort declined significantly during the same period, dropping from 575 to 501 patients per 100,000 (*p* < 0.0001). Similarly, PD incidence decreased from 69 to 60 patients per 100,000 (*p* = 0.0001).

**Conclusions:**

PD prevalence increased in the study population but declined in the extrapolated cohort, while incidence remained stable overall in the study population, yet decreased in the extrapolated cohort. This study provides key insights into the epidemiology of PD in Germany, highlighting the influence of methodology and the need for ongoing monitoring to inform healthcare planning.

## Graphical abstract

This cross‐sectional claims data analysis provides insights into the epidemiology of Parkinson's disease in Germany. Prevalence showed inconsistent trends, while temporary incidence reductions occurred during the COVID‐19 pandemic. Average age of patients increased, highlighting the continued disease burden and need for ongoing monitoring, informing healthcare planning in aging societies.

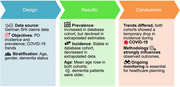

## 1 Introduction

Parkinson's disease (PD) is a progressive neurodegenerative disorder marked by dopamine deficiency and neuronal loss in the substantia nigra, leading to motor symptoms such as bradykinesia, tremor, rigidity, and postural instability, which cause significant disability. Non‐motor symptoms—including hyposmia, constipation, depression, and sleep disturbances—often precede motor signs. Cognitive impairment and dementia are also common features of PD, and at least 75% of patients who live beyond 10 years develop dementia. The exact cause of PD remains unclear, but it is generally accepted that a combination of genetic, environmental, and lifestyle factors contributes to its pathogenesis (Balestrino and Schapira 2020; Poewe et al. 2017; Simon et al. 2020; Sung and Nicholas 2013; Wattenbach et al. 2024).

PD is the fastest growing neurological disease worldwide in terms of prevalence, disability, and mortality (Dorsey et al. 2018; GBD Parkinson's Disease Collaborators 2018; GBD Neurological Disorders Collaborator Group 2017). Globally, PD prevalence varies widely, from ∼100 per 100,000 in East Asia to over 1500 per 100,000 in older North American populations (Park et al. 2019; Willis et al. 2022). The global burden of PD is rising rapidly, with 11.8 million cases in 2021 projected to reach 25.2 million by 2050, driven primarily by population aging and growth (Luo et al. 2024), placing increasing pressure on health systems (Wanneveich et al. 2018; Kowal et al. 2013).

In Europe, prevalence estimates range from 65 to 1200 per 100,000 (von Campenhausen et al. 2005), while in Germany, recent studies report ∼200–1000 per 100,000, depending on population and methodology (Wattenbach et al. 2024; Nerius et al. 2017; Dammertz et al. 2023).

Incidence rates show similar variability. Globally, rates range from ∼20 to > 100 per 100,000 (Park et al. 2019; Willis et al. 2022). In Europe, most primary incidence data are outdated, though a recent population‐based study from Norway provides robust estimates (Brakedal et al. 2022). Modeled GBD 2021 estimates indicate ∼40 per 100,000 in Western Europe, with Germany among the highest at ∼53 per 100,000. German studies report conflicting results: 192–229 per 100,000 (Nerius et al. 2017), 137–106 per 100,000 (2013–2019) (Dammertz et al. 2023), and substantially higher incidence in older adults (Wattenbach et al. 2024). Together, these findings demonstrate the lack of contemporary, reliable epidemiological data for Germany and underscore the need for updated national estimates.

This study aims to estimate PD incidence and prevalence within a subpopulation of the German statutory health insurance (SHI) system from 2016 to 2021, assess trends during the COVID‐19 pandemic, and provide a demographic analysis of PD patients by age, gender, and dementia, as these factors are known to influence disease epidemiology and clinical course.

## 2 Methods

### 2.1 Data Acquisition

Secondary data from the Scientific Institute for Health Economics and Health Systems Research (WIG2) (Wissenschaftliches Institut für Gesundheitsökonomie und Gesundheitssystemforschung [WIG2 GmbH] n.d.) were used for this study.

In Germany, in line with the Good Practice of Secondary Data Analysis (GPS), no formal ethical approval is required as no primary collection of individual human data occurred, and only anonymized healthcare data were used.

### 2.2 Database Description

The present study is a cross‐sectional claims data analysis for January 2016–2021. The data are sourced from the German SHI system, covering 89.35% of the overall population in Germany (GKV‐Spitzenverband n.d.), with the WIG2 database covering approximately 6% of the total SHI population and, as such, considered representative of the German population.

The WIG2 database is an anonymized healthcare claims database with longitudinal data from approximately 4.5 million Germans insured by one of various German SHIs and contains data from the beginning of 2010 to the end of 2021 with a low attrition rate, providing a longitudinal observation of maximum 11 years. It includes core data on insured persons and full billing information of utilized health services in hospitals, the ambulatory sector, and pharmaceuticals. The representativeness of these data in terms of age, sex, and morbidity distribution is given for the entire SHI‐insured population (Ständer et al. 2020). Before being entered into the WIG2 database, the data are anonymized with respect to individual insurance, healthcare providers (e.g., physicians, practices, hospitals, and pharmacies), and the respective SHI. After application of inclusion/exclusion criteria (described below), approximately 2.5 million persons, including 8500 persons with PD, were available in the WIG2 database per study year.

### 2.3 Study Cohorts

The inclusion criteria of the analysis, as well as definitions for inclusion within the prevalent and incident PD population are included in **Table** **1**. The total observation period of the study focused on the years 2016–2021.

**TABLE 1:** Inclusion criteria for the analysis population.

**Inclusion criteria**
1 At least 1 day of insurance in the research database in a study year
2 At least 18 years of age
3 Continuous insurance for the whole study period or death during the study period
4 Continuous insurance for 1 year preceding the study year (baseline period)
**Prevalent population identification criteria**
One main inpatient discharge diagnosis and/or two confirmed outpatient diagnoses in different quarters Secondary inpatient diagnoses are treated as confirmed outpatient diagnoses To classify a patient as having PD, the M2Q criterion is applied to the timeframe of one calendar year (i.e., study year), with the second confirmed outpatient diagnosis or secondary inpatient diagnoses documented in a different quarter, but within the same calendar year as the first PD diagnosis
**Incident population identification criteria**
One main inpatient discharge diagnosis and/or two confirmed outpatient diagnoses in different quarters. Secondary inpatient diagnoses are treated as confirmed outpatient diagnoses. To classify a patient as having PD, the M2Q criterion is applied to the timeframe of one calendar year (i.e., study year), with the second confirmed outpatient diagnosis or secondary inpatient diagnoses documented in a different quarter, but within the same calendar year as the first PD diagnosis No confirmed outpatient or inpatient diagnosis for PD in the year prior to the study year (baseline period)

*Note*: M2Q criterion: This criterion indicates reimbursement of at least one contact to the practice due to a certain condition in at least two quarters of the last 12 months (Naing 2000). ICD codes used for patient identification are provided in the Supporting Information Appendix.

Abbreviation: PD, Parkinson's disease.

A sensitivity analysis was conducted by assessing differences between the PD population “with” and “without” a previous history of dementia.

Prevalence and incidence data from the WIG2 database were subsequently extrapolated to represent the full SHI population; henceforth, this group will be referred to as the “extrapolated cohort.” Age‐ and sex‐standardized rates were calculated using the method of direct standardization (Naing 2000), based on the KM6 statistics (Bundesministerium für Gesundheit [BMG] n.d.) of the respective year as the standard population.

### 2.4 Endpoints

Prevalence and incidence were expressed as patients per 100,000 persons per year for the main cohort and the non‐dementia and dementia subgroups. For both prevalence and incidence, the following endpoints were reported for the main cohort and the two subgroups:

- Percentage age distribution per year, using 5‐year groupings from 65 to ≥ 90 years group.
- Average age by gender, presenting overall, female, and male groups per year and cohort.

### 2.5 Statistical Analysis

All analyses were conducted on a person level and not on a case level. Hence, there was no double counting as well as no clustering within an individual observation year. Patients were included in the analysis for each year in which a PD diagnosis was documented.

The cohort was stratified by dementia status, age, and gender to account for differences in disease course, comorbidities, healthcare use, and demographic influences, enabling more precise epidemiological analyses and minimizing confounding by cognitive or demographic factors.

The proportion of PD patients was measured in the database. Statistical testing of differences in the PD prevalence and incidence was conducted using normal approximation to estimate confidence intervals and *p*‐values. A trend analysis using a univariate linear regression was conducted to estimate the trend in prevalence and incidence between 2016 and 2021. Year of observation was used as independent variable and the yearly prevalence/incidence rate as dependent variable. The regression coefficient and Wald confidence interval are displayed as estimates for the trend. Data preparation was done using MS SQL Server. R 4.2 was used for regression analysis.

## 3 Results

### 3.1 Prevalence Increase in WIG2 Database

**FIGURE 1:**
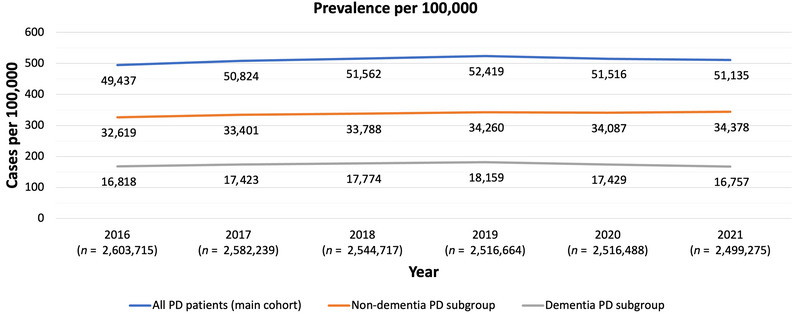
Prevalence of PD (WIG2 database) in Germany for the years 2016–2021 for all PD patients (main cohort), non‐dementia PD subgroup, and dementia PD subgroup. *n*, total number of eligible persons at risk in WIG2 database in study year; PD, Parkinson's disease.

**FIGURE 2:**
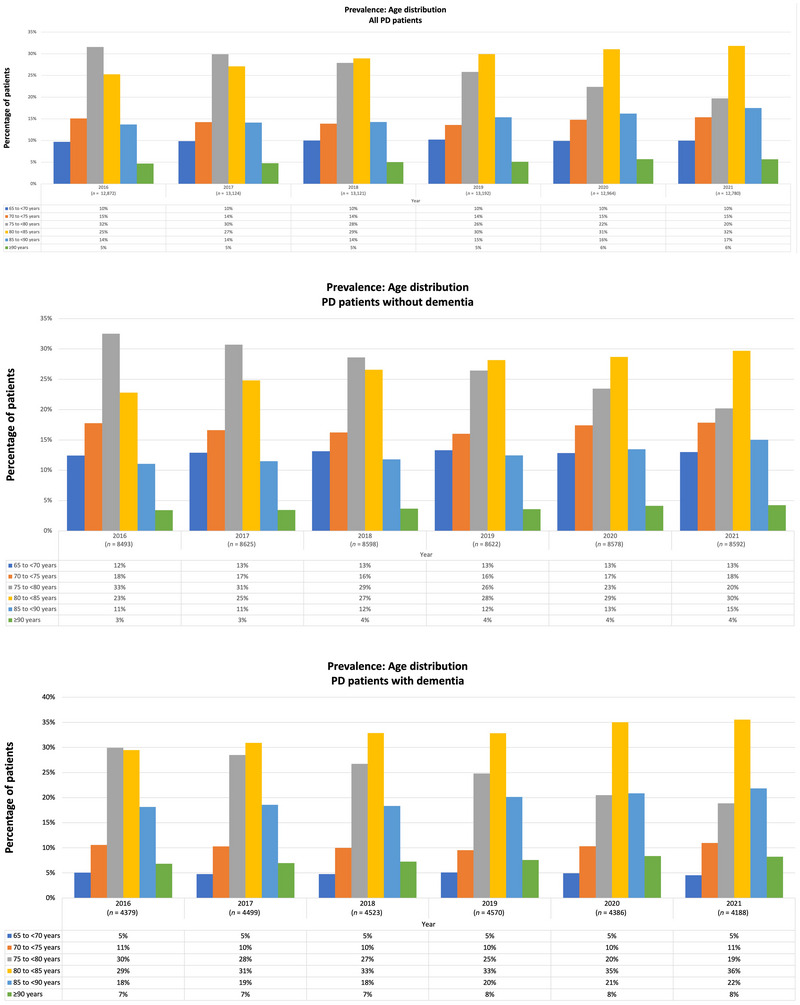
Distribution of age groups in the prevalent PD population (WIG2 database) in Germany for the years 2016–2021 for (A) all PD patients, (B) PD patients without dementia, and (C) PD patients with dementia. *n*: PD patients in WIG2 database per year; PD: Parkinson's disease.

The total number of eligible individuals in the WIG2 database was 2,603,715 in 2016, decreasing to 2,499,275 in 2021 (Figure 1). Of these, approximately 12,800–13,200 individuals (Figure 2A) met the inclusion criteria for prevalence in each assessed year, representing the population of persons with PD analyzed in this study.

The overall PD prevalence in the assessed population increased significantly by 17 from 494 patients per 100,000 in 2016 to 511 in 2021 (Figure 1) (*p* = 0.0067 [95% CI 4.7, 29.25]) (Table S1). For persons with PD without dementia, the prevalence rose significantly from 326 patients per 100,000 in 2016 to 344 in 2021 (*p* = 0.0006). Prevalence of people with PD and dementia fluctuated from 2016 to 2021 without significant changes (Table S1).

Rolling 2‐year comparisons showed no significant changes, except for the overall PD prevalence in 2017 compared to 2016 (*p* = 0.0253) (Table S1).

### 3.2 Prevalence Decrease in Extrapolated Cohort

In contrast, the total number of eligible individuals in the extrapolated cohort increased by 36,762 from 60,257,947 in 2016 to 60,294,709 in 2021. Of these, approximately 300,000 individuals per year met the inclusion criteria for PD prevalence (data not shown).

Unlike the WIG2 prevalent population, PD prevalence in the extrapolated cohort declined significantly across all three analyzed cohorts between 2016 and 2021: overall PD prevalence decreased by 74 from 575 patients per 100,000 to 501 (*p* < 0.0001; 95% CI: −86.93, −61.55); for PD patients without dementia, prevalence fell from 368 to 335 per 100,000 (*p* < 0.0001); and for PD patients with dementia, prevalence dropped from 207 to 167 per 100,000 (*p* < 0.0001) (Table S2).

### 3.3 Prevalent Age Increase in WIG2 Database

**FIGURE 3:**
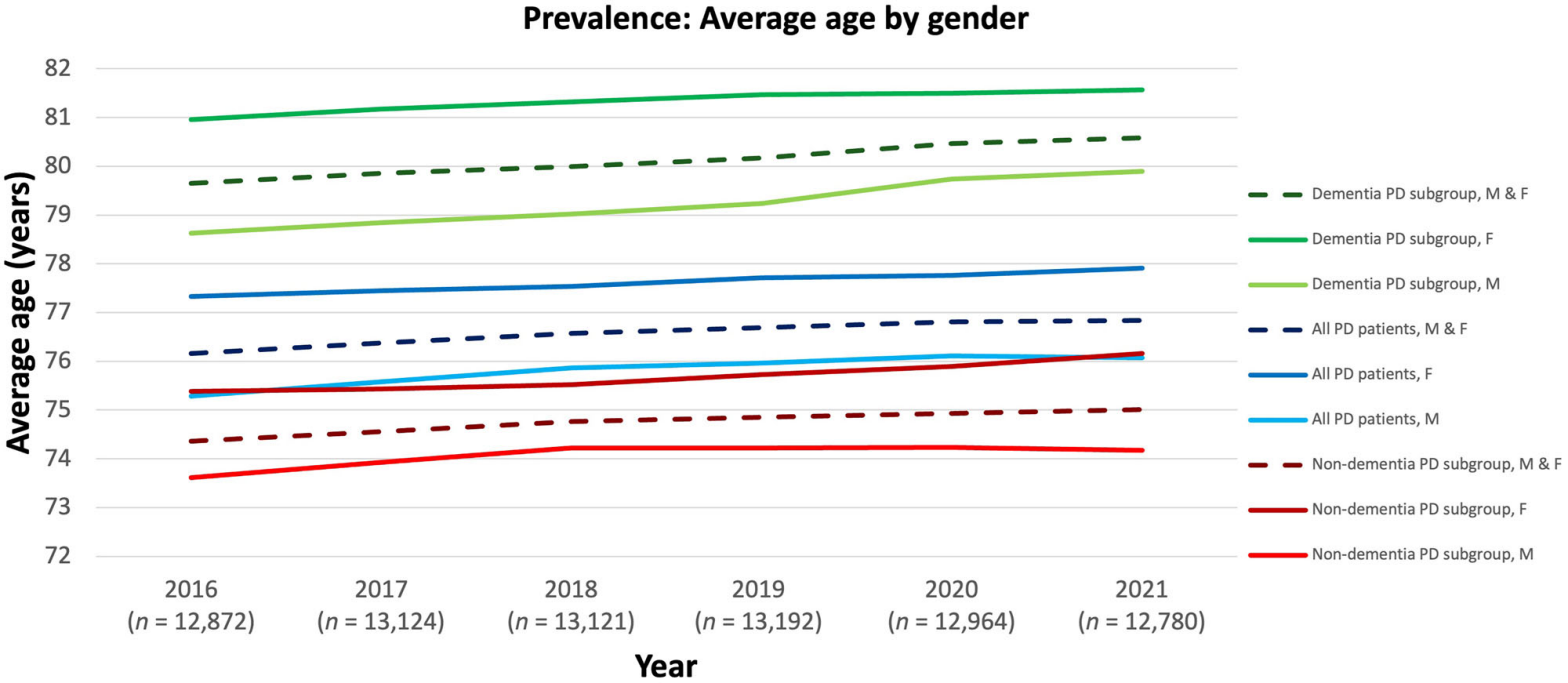
Average age of prevalent PD patients (WIG2 database) in Germany for the years 2016–2021 split by gender for all PD patients (main cohort), non‐dementia PD subgroup, and dementia PD subgroup. F: female; M: male; *n*: PD patients in WIG2 database per year; PD: Parkinson's disease.

The average age of PD patients in the prevalent WIG2 population was 76.6 years. PD patients with a history of dementia were older, averaging 80.1 years, while those without dementia averaged 74.8 years. The majority of persons with PD in Germany were aged ≥ 75, with a steady increase in the ≥ 80 age group from 2016 to 2021 (Figure 2A–C). The largest age group (regardless of dementia status) in 2016 was 75–< 80 years, experiencing a decline over time, alongside an increase in the 80–<85 age group. Between 2016 and 2021, the average age of people with PD increased by 7.5 months, a trend consistent across all three cohorts. Women were generally 2 years older than men (Figure 3). Additionally, more PD patients were recorded as male than as female (data not shown).

### 3.4 Incidence Increase in WIG2 Database

**FIGURE 4:**
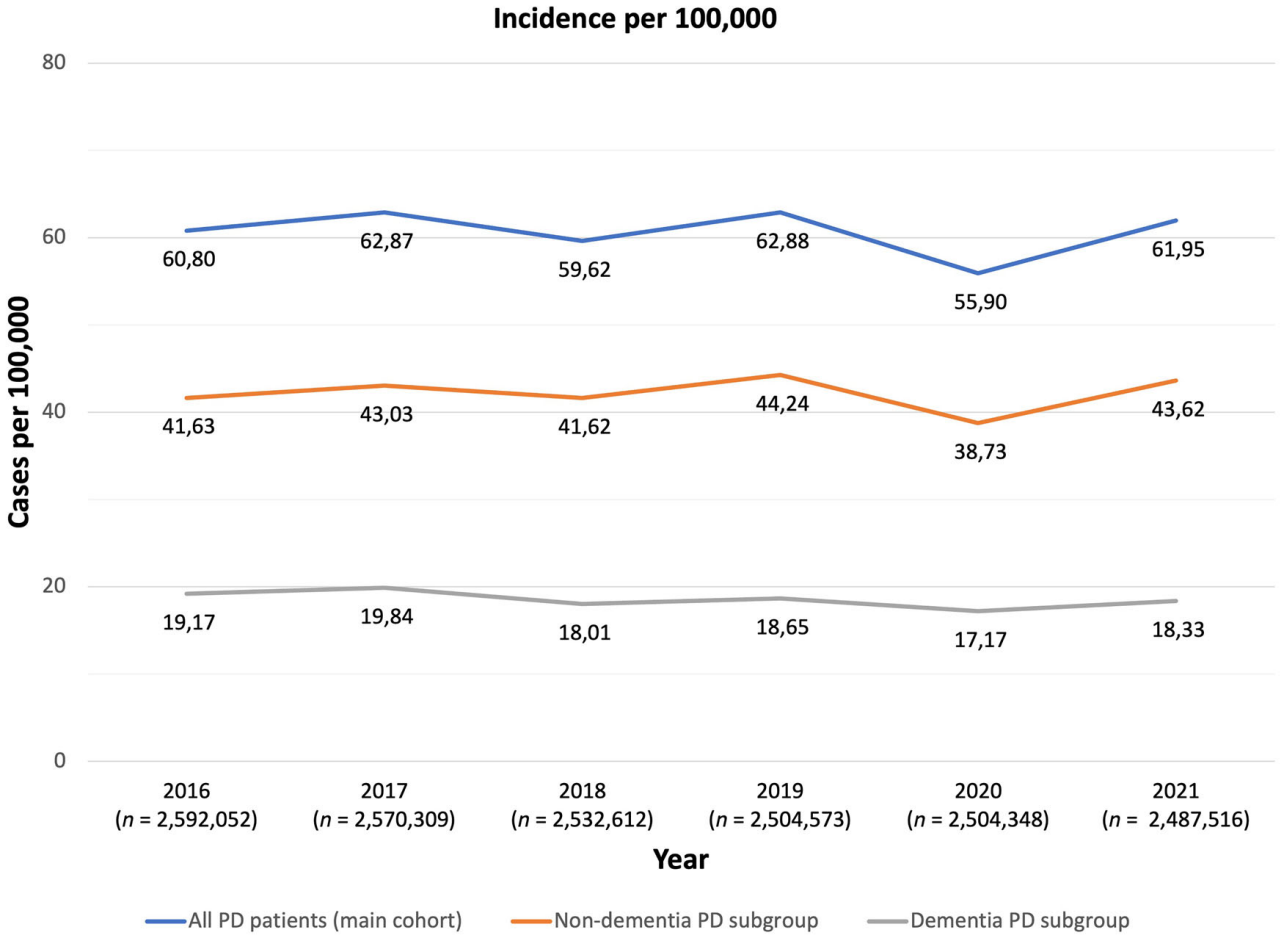
Incidence of PD (WIG2 database) in Germany for the years 2016–2021 for all PD patients (main cohort), non‐dementia PD subgroup, and dementia PD subgroup. *n*: Total number of eligible persons at risk in WIG2 database in study year; PD: Parkinson's disease.

**FIGURE 5:**
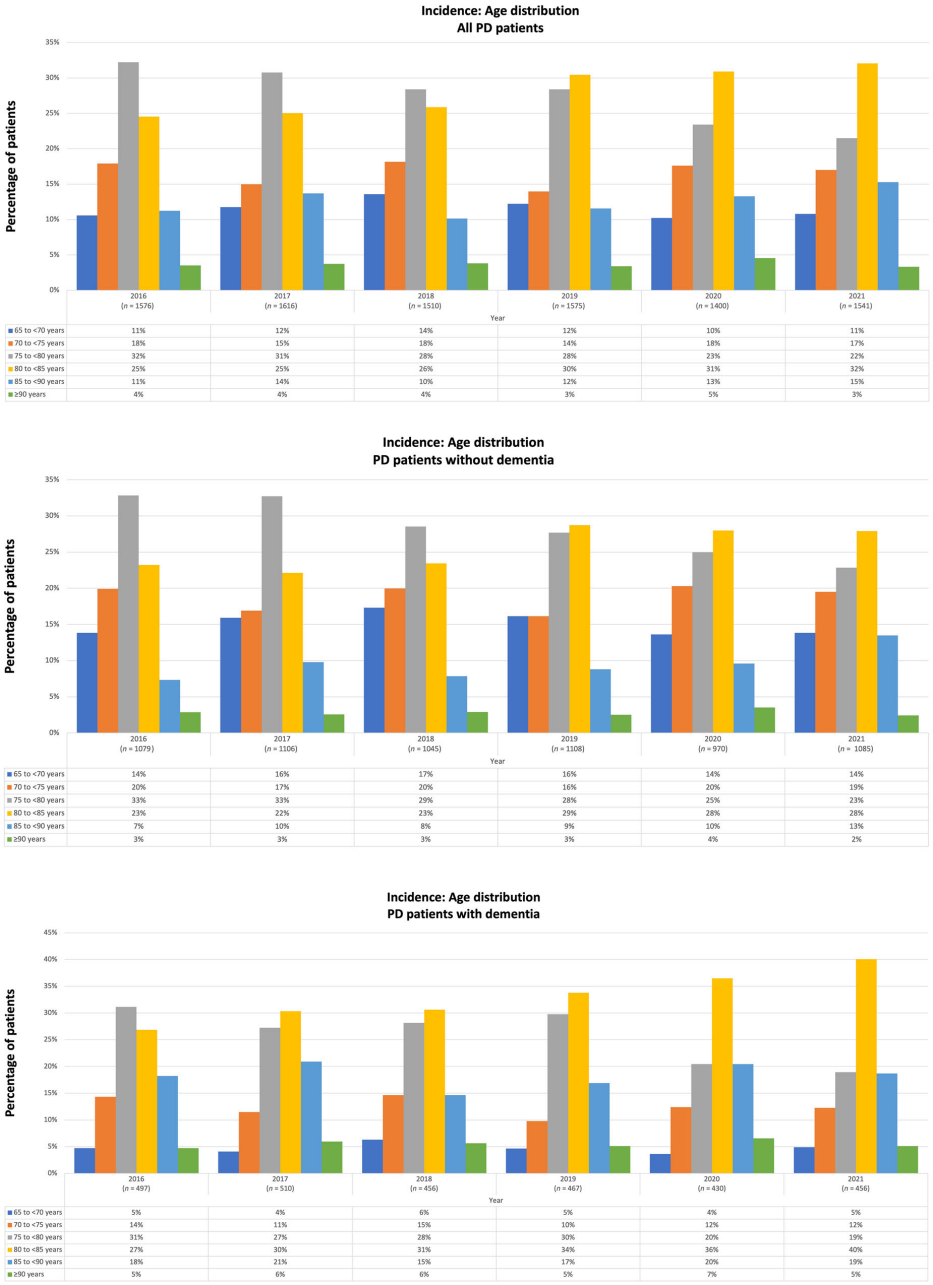
Distribution of age groups in the incident PD population (WIG2 database) in Germany for the years 2016–2021 for (A) all PD patients, (B) PD patients without dementia, and (C) PD patients with dementia. n: PD patients in WIG2 database per year; PD: Parkinson's disease.

For these analyses, the total number of eligible individuals in the WIG2 database decreased from 2,592,052 in 2016 to 2,487,516 in 2021 (Figure 4). Of these, approximately 1500 individuals per year (Figure 5A) met the inclusion criteria for incidence, representing the incident PD population examined in this study.

In the overall cohort and for PD patients without dementia, incidence increased numerically, but not significantly between 2016 and 2021 (Figure 4). In contrast, incidence of persons with PD and dementia decreased over the same period, but again, these changes were not statistically significant (Table S3).

In 2020, PD incidence rates fell significantly by 7 from 62.9 patients per 100,000 to 55.9 in all persons with PD (*p* = 0.0013 [95% CI −11.25, −2.71]), and from 44.2 patients per 100,000 to 38.7 for PD patients without dementia (*p* = 0.0025). Similar changes in the PD with dementia subgroup were not significant (Table S3).

In 2021, incidence rates rebounded significantly by 6–62.0 patients per 100,000 in all persons with PD (*p* = 0.0054; 95% CI: 1.79, 10.30) and to 43.6 patients per 100,000 in the non‐dementia PD subgroup (*p* = 0.0072), while changes in the PD with dementia subgroup remained non‐significant (Table S3).

### 3.5 Incidence Decrease in Extrapolated Cohort

The number of eligible individuals in the extrapolated cohort increased by 75,066, from 59,940,997 in 2016 to 60,016,063 in 2021. Approximately 33,000–41,000 individuals per year met the incidence inclusion criteria (data not shown).

Unlike the WIG2 cohort, PD incidence in the SHI population decreased across all analyzed cohorts between 2016 and 2021: Incidence in all persons with PD fell by 8.5 from 68.85 to 60.30 patients per 100,000 (*p* = 0.0001; 95% CI: −12.97, −4.14); for PD patients without dementia from 45.74 to 42.41 (*p* = 0.0745); and in PD patients with dementia from 23.12 to 17.89 (*p* < 0.0001) (Table S4).

In 2020, incidence rates dropped significantly overall by 7.6 from 63.39 to 55.80 (*p* = 0.0005; 95% CI: −11.86, −3.31); for non‐dementia PD patients from 44.00 to 38.79 (*p* = 0.0042); and in the PD with dementia subgroup from 19.39 to 17.01 (*p* = 0.0485) (Table S4).

In 2021, incidence rebounded significantly by 4.5–60.30 overall (*p* = 0.0370; 95% CI: 0.27, 8.72) and to 42.41 in the non‐dementia PD subgroup (*p* = 0.0445), while changes in the PD with dementia subgroup were not significant (Table S4).

### 3.6 Incident Age Increase in WIG2 Database

In the overall incident PD population, 72% of patients were aged ≥ 75 in 2016 with the proportion of patients aged ≥ 80 increasing from 40% in 2016 to 50% in 2021 (Figure 5A). The incident population also showed a general trend of increasing age during this period. In 2016, the largest age group (regardless of dementia status) was 75–< 80 years, whereas by 2021, the 80–< 85 age group became the largest (Figure 5A–C). In 2021, 40% of newly diagnosed PD patients with a history of dementia were in the 80–< 85 age group, and 24% were ≥ 85 years old (Figure 5C).

**FIGURE 6:**
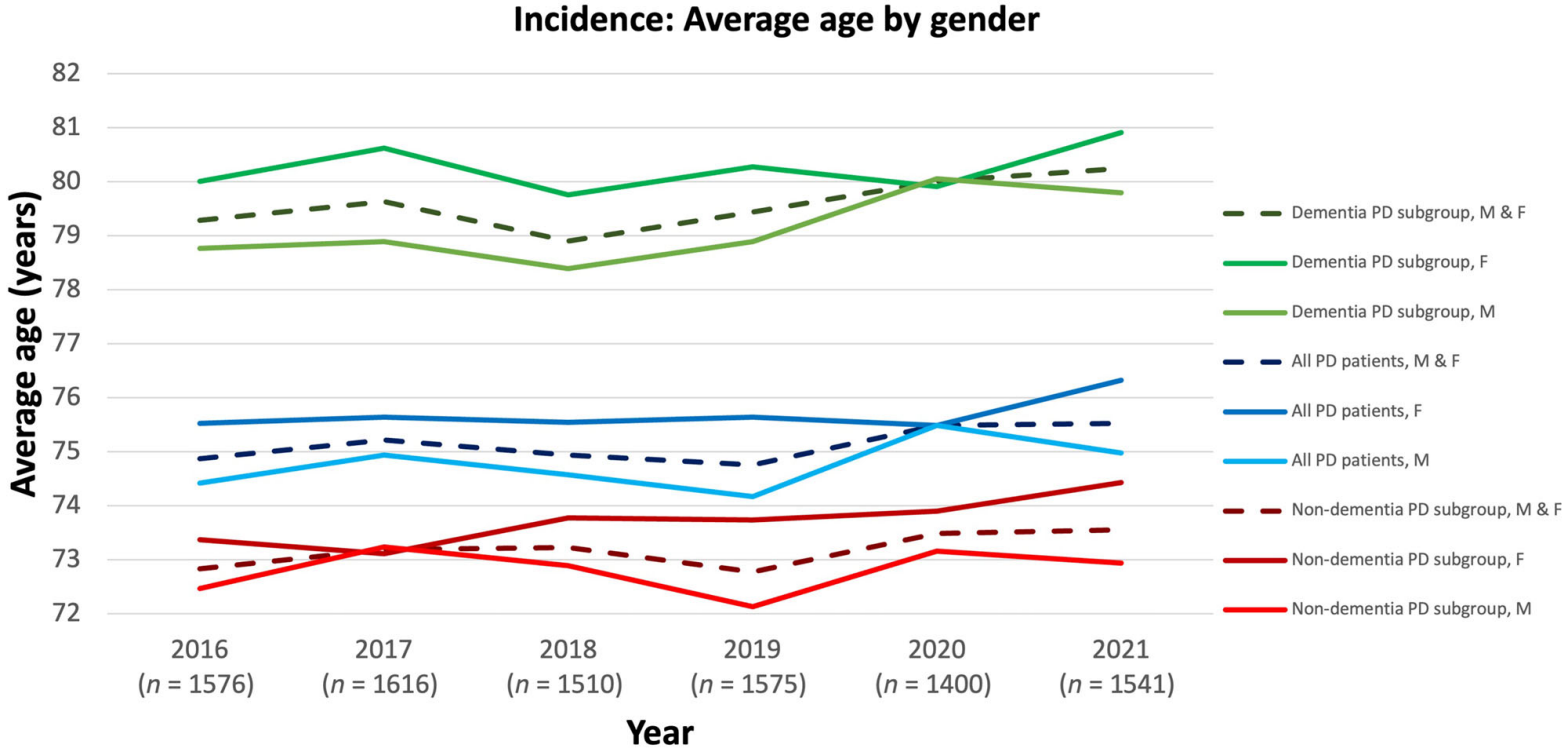
Average age of incident PD patients (WIG2 database) in Germany for the years 2016–2021 split by gender for all PD patients (main cohort), non‐dementia PD subgroup, and dementia PD subgroup. F: female; M: male; *n*: PD patients in WIG2 database per year; PD: Parkinson's disease.

From 2016 to 2021, the average age of newly diagnosed PD patients increased by 7.5 months. This was the same in the dementia PD population, who presented with the highest overall age. As demonstrated in the prevalent PD population, women in the incident group were generally 0.5–1.5 years older than men (Figure 6), with more patients recorded as male than as female (data not shown).

## 4 Discussion

### 4.1 Summary

This study provides population‐representative insights into incidence and prevalence of PD in Germany between 2016 and 2021, using WIG2 claims data and extrapolated estimates for the SHI population. Three cohorts were analyzed: all PD patients, and subgroups with and without dementia.

### 4.2 Prevalence in WIG2 Database

Between 2016 and 2021, PD prevalence increased in all three analyzed cohorts. The overall prevalence of PD in Germany grew by 3% over the 6 years assessed. This increase was driven by a 5% growth in prevalence of PD patients without dementia. The absolute PD prevalence in 2021 was 511.35 patients per 100,000. Of these, approximately 344 patients were individuals without dementia, and 170 patients had a history of dementia. These figures are consistent with previously reported estimates, such as the 2021 PD prevalence of 494.2 per 100,000 reported by the Institute for Health Metrics and Evaluation (IHME Global Burden of Disease 2024). Similar numbers have also been published in a range of other analyses (Heinzel et al. 2018).

The significant growth in PD prevalence observed in the present WIG2 analysis aligns with earlier findings. A similar upward trend was observed in Norway, where PD prevalence rose across nearly all age groups between 2005 and 2016, with the highest relative increases observed in individuals aged ≥ 70 years (Brakedal et al. 2022). Furthermore, other studies have shown similar increases (GBD Parkinson's Disease Collaborators 2018; Pupillo et al. 2016). In addition, a recent analysis from the GBD Study 2021 further highlights the growing global PD burden. The global PD prevalence is projected to increase by 112% by 2050, reaching 267 (230–320) cases per 100,000 persons (Luo et al. 2024).

The rising prevalence of PD without dementia increasingly strains ambulatory neurology services, caregiver support, and rehabilitation programs focused on maintaining function and quality of life (Poewe et al. 2008). Although no disease‐modifying therapy (DMT) has yet been approved, several late‐stage candidates may hold promise to slow disease progression, which could further intensify demand on outpatient care (Bloem et al. 2021). To address these challenges, coordinated strategies involving Parkinson's networks, healthcare providers, and policymakers are essential to ensure sustainable and patient‐centered management.

### 4.3 Prevalence in Extrapolated Cohort

The extrapolated dataset in the current analysis indicated a notable decline in PD prevalence across all three cohorts. This could be explained by increased immigration of younger individuals (without PD) into Germany, leading to an expansion in the overall German (and SHI) population. At the same time, a bulge in the German population, caused by increased birthrates in the years after WWII, known as the “baby boomer” population, is starting to reduce in size due to age‐driven mortality, leading to a reduction in the number of people with PD. These two factors have likely led to a lower ratio of PD patients to healthy individuals in the German population. This trend was also observed by Rommel et al. (2025), who reported a decrease in age‐standardized PD prevalence from 0.38% in 2017 to 0.29% in 2022. Bohlken et al. (2022) also reported a decline in the number of PD patients between 2010 and 2019 from 3352 (0.36% of all patients) to 3541 (0.33%) in German general practitioner practices.

Interestingly, in the present study we extrapolated that in 2016 there were 346,683 persons with PD living in Germany, which declined to 302,130 by 2021. These numbers differ starkly from a previous analysis carried out in 2018 that indicated PD prevalence in Germany was only 162,246 (GBD Parkinson's Disease Collaborators 2018). This highlights the potential variability in using prevalence rates to calculate absolute prevalence.

### 4.4 Incidence in WIG2 Database

Between 2016 and 2021, PD incidence in the WIG2 database remained largely stable. However, a significant decrease in incidence occurred across all PD cohorts between 2019 and 2020 (−1.5 to −7/100,000), likely reflecting reduced diagnostic activity during the COVID‐19 pandemic. Incidence rebounded in 2021, but not to pre‐pandemic levels, suggesting that true incidence may have been unaffected. Instead, PD diagnosis rates may have been transiently reduced during the period of increased anti‐infection measures, reduced clinical touchpoints, and reduction of non‐essential services that occurred during the COVID‐19 pandemic. However, one would have expected a more pronounced increase in diagnosis in subsequent years as the backlog of undiagnosed persons with PD were processed and diagnosed. Yet, no clear compensatory increase was observed post‐pandemic, indicating that any diagnostic backlog may not have been fully resolved. Another possible factor is that some individuals who would have been newly diagnosed with PD may have died from COVID‐19 before diagnosis, contributing to the observed decrease in incidence and lack of a compensatory increase post‐pandemic.

### 4.5 Incidence in Extrapolated Cohort

In the extrapolated dataset, a significant decrease in incident PD patients was shown in the overall PD population and in PD patients with dementia between 2016 and 2021. The most marked decrease in incidence occurred during 2020, which coincided with the COVID‐19 pandemic, and as with the WIG2 database data, incidence rates failed to recover to pre‐COVID levels in 2021.

Although PD is not generally seen as a risk factor for COVID‐19 infection, PD patients likely self‐isolated more than the general population due to social distancing, contact restrictions, and fear of infection (Fearon and Fasano 2022). This likely led to reduced health service use and postponed appointments (Fasano et al. 2020; Feeney et al. 2021; Salari et al. 2020; Zipprich et al. 2020), possibly resulting in fewer PD diagnoses in 2020. Furthermore, the reduction in PD incidence demonstrated here is not unprecedented, as Anne et al. (2023) showed an overall decrease in the German age‐specific PD incidence rates over a 10‐year period (2004–2009 and 2014–2019) in both sexes, even after controlling for sex, PD stage, risk factors, comorbidities, and changes in death rates. Similarly, Dammertz et al. (2023) reported a decline in idiopathic PD incidence in Germany from 137 to 106 per 100,000 between 2013 and 2019 across all demographics. However, in an official response to the study by Dammertz et al., the German Society for Parkinson's Disease and Movement Disorders (DPG) questioned the findings, stating that PD incidence remains high and no trend reversal is expected (Deutsche Gesellschaft für Parkinson und Bewegungsstörungen e.V. [DPG] 2022). This highlights conflicting views on PD incidence globally, with some regions anticipating a PD crisis. Variability in incidence estimates stems from differences in data sources, methods, definitions, and observation periods. Recent analyses, including this one, suggest earlier projections of steep increases in PD incidence and prevalence may be overstated (Wanneveich et al. 2018).

### 4.6 Age and Gender Distribution

The average age of PD patients in the WIG2 database was 75–77 years in both the incident and prevalent populations. However, again, this was not a stable statistic, and not only did the average age of PD patients in Germany increase regardless of dementia status during the assessment period, but the largest age group shifted from the 75 to < 80 age group in 2016 to the 80 to < 85 age group by 2021, regardless of dementia status. One explanation for this trend is the overall decline in mortality from other causes in aging populations, which results in a larger number of individuals being at risk for developing neurodegenerative diseases as age increases (Ben‐Shlomo et al. 2024). Similarly, PD patients with dementia were generally older than patients in the overall PD population, reflecting the general trend that dementia risk increases with age (Olfson et al. 2021; Kramarow 2024).

These findings are consistent with published literature with numerous studies citing higher PD prevalence and incidence with increasing age and male gender via various methods (Rommel et al. 2025). These studies also showed that women generally had a higher age than men across all cohorts (Wattenbach et al. 2024). These findings are consistent with data from other countries (Willis et al. 2022; Brakedal et al. 2022; Pupillo et al. 2016; Blin et al. 2015).

Given the cross‐country comparisons presented above, it is important to consider that differences in epidemiological estimates may also be influenced by methodological heterogeneity, including variations in data sources, case definitions, and health system structures. Previous work has shown that such factors, for example, differences between claims data and registry sources, can substantially affect estimates and limit comparability across settings (Dorsey et al. 2018; Nerius et al. 2017; Kreis et al. 2016). While these issues are highly relevant, a more detailed discussion is beyond the scope of the present study.

## 5 Conclusion

In conclusion, this analysis demonstrated that between 2016 and 2021 in Germany:

1. Prevalence in the WIG2 database cohort increased, while extrapolated SHI estimates declined, reflecting differences in methodology.
2. Incidence in the WIG2 database remained stable but declined in extrapolated data, with both cohorts showing temporary reductions during the COVID‐19 pandemic.
3. The average age of individuals with PD increased in both incident and prevalent populations. Patients with dementia were typically older than those without, and males with PD were generally younger than females across all cohorts.

### 5.1 Limitations

This study has several limitations. First, the analysis relies on claims data from a subset of the SHI system, excluding privately insured individuals who represent about 10% of the German population. Although this may introduce a certain selection bias, as privately insured individuals often differ in socioeconomic status and care patterns, the dataset remains robust, representing the vast majority of the German population. Importantly, since health insurance is mandatory in Germany, all residents are insured and have direct access to specialist care, including neurologists, if required.

Second, diagnoses are based on ICD coding, which introduces potential misclassification bias and may lead to under‐ or overestimation of the analyzed population depending on coding practices. Although the M2Q algorithm improves specificity, it cannot fully correct for diagnostic inaccuracies or variability. For example, persons with PD typically develop dementia.

In later stages, so dementia present at diagnosis may reflect secondary or atypical forms of PD. Such patterns may also result from coding practices within the SHI, where data are primarily collected for reimbursement purposes, potentially contributing to misclassification bias in incidence and prevalence analyses.

Third, the potential underassessment of PD during the COVID‐19 pandemic may have influenced observed incidence and prevalence patterns. Although reduced diagnostic activity and healthcare disruptions during 2020 likely contributed to the temporary decline in diagnoses, it remains uncertain to what extent these factors versus other epidemiological influences, such as excess mortality among undiagnosed individuals, affected the observed rates.

Fourth, while data on treatment and non‐dementia comorbidities were collected, these variables are not reported here, and the study therefore cannot provide detailed insights into these aspects of the disease.

Lastly, some data were produced by extrapolation. Although recognized and pre‐published methods were utilized for extrapolation, there was little scope to test the variable effect of using different extrapolation methods or making comparisons to data taken from the full SHI population. As such, this study cannot provide a definitive narrative as to the current prevalence and incidence rates of PD across all of Germany but instead provides robust evidence for changes across the WIG2 population with an indication of how these outcomes could relate to the total German SHI population.

### 5.2 Areas for Further Research

Future research should examine PD incidence and prevalence beyond 2021, with a focus on factors influencing disease progression and on evaluating the real‐world impact of emerging therapies. Importantly, these studies should be designed to help healthcare systems and providers anticipate and prepare for the practical implications of new treatments. In addition, investigations into variations in diagnosis, treatment patterns, and gender‐specific outcomes will be essential to support patient‐centered management and guide healthcare planning as the PD population grows.

## Author Contributions

**Dirk Woitalla**: writing – review and editing (equal). **Gisa Ellrichmann**: writing – review and editing (equal). **Stefan Jun Groiss**: writing – review and editing (equal). **Stefan Walzer**: writing – original draft preparation (equal); writing – review and editing (equal). **Lutz‐Michael Vollmer**: writing – original draft preparation (equal); writing – review and editing (equal). **Signe Thiesen‐Nash**: conceptualization (equal); writing – review and editing (equal). **Sebastian Krenberger**: writing – original draft preparation (lead); writing – review and editing (equal). **Julia Borchert**: conceptualization (equal); formal analysis (lead); methodology (lead). **Glynn Harrison‐Jones**: conceptualization (equal); formal analysis (equal); writing—review and editing (lead).

## Ethics Statement

In Germany, in line with the Good Practice of Secondary Data Analysis (GPS), no formal ethical approval is required as no primary collection of individual human data occurred, and only anonymized healthcare data were used.

## Funding

This work was funded by BIAL – Portela & Ca, S.A.

## Conflicts of Interest

Dirk Woitalla and Stefan Jun Groiss declare that they have not received any financial compensation for the publication of this paper. However, in the past 2 years, they have each accepted speaker fees for educational events and have each served as consultants on advisory boards for BIAL.

## Peer Review

The peer review history for this article is available at https://publons.com/publon/10.1002/brb3.71027.

## Supporting information




**Supplementary Tables**: brb371027‐sup‐0001‐tableS1‐S4.docx

**Supplementary Material**: brb371027‐sup‐0002‐appendix.docx

## Data Availability

The data that support the findings of this study are available from the corresponding author upon reasonable request.
