# Supplementary material for: Epidemiology of Parkinson's Disease in Germany Between 2016 and 2021 Based on Statutory Health Insurance Claims Data
Source: Brain Behav. 2025 Nov 5;15(11):e71027. doi: 10.1002/brb3.71027 (PMC12589807; doi:10.1002/brb3.71027)
Supplement: Supplementary file 1 — Supplementary Tables: brb371027‐sup‐0001‐tableS1‐S4.docx [file BRB3-15-e71027-s002.docx]

**Table 1: Rolling 2-year comparisons and 5-year^#^ comparison of prevalent PD cases in Germany from the WIG2 database**

| **Year** | **Prevalence** | **Prevalence change to previous year** | **Lower CI** | **Upper CI** | **P value** |
| --- | --- | --- | --- | --- | --- |
| *All PD patients* | | | | | |
| **2016** | 494.37 |  | | | |
| **2017** | 508.24 | 13.87 | 1.71 | 26.03 | 0.0253* |
| **2018** | 515.62 | 7.38 | -4.98 | 19.73 | 0.2419 |
| **2019** | 524.19 | 8.57 | -3.96 | 21.10 | 0.1802 |
| **2020** | 515.16 | -9.02 | -21.59 | 3.54 | 0.1592 |
| **2021** | 511.35 | -3.81 | -16.32 | 8.69 | 0.5500 |
| **2016 – 2021*^#^*** |  | 16.98^#^ | 4.70^#^ | 29.25^#^ | 0.0067* |
| *PD patients without a history of dementia* | | | | | |
| **2016** | 326.19 |  | | | |
| **2017** | 334.01 | 7.82 | -2.05 | 17.70 | 0.1204 |
| **2018** | 337.88 | 3.86 | -6.15 | 13.88 | 0.4497 |
| **2019** | 342.60 | 4.72 | -5.43 | 14.87 | 0.3619 |
| **2020** | 340.87 | -1.72 | -11.92 | 8.47 | 0.7403 |
| **2021** | 343.78 | 2.91 | -7.32 | 13.13 | 0.5772 |
| **2016 – 2021*^#^*** |  | 17.59^#^ | 7.56^#^ | 27.62^#^ | 0.0006* |
| *PD patients with history of dementia* | | | | | |
| **2016** | 168.18 |  | | | |
| **2017** | 174.23 | 6.05 | -1.07 | 13.16 | 0.0959 |
| **2018** | 177.74 | 3.51 | -3.74 | 10.77 | 0.3428 |
| **2019** | 181.59 | 3.85 | -3.53 | 11.23 | 0.3066 |
| **2020** | 174.29 | -7.30 | -14.66 | 0.07 | 0.0521 |
| **2021** | 167.57 | -6.72 | -13.95 | 0.51 | 0.0684 |
| **2016 – 2021*^#^*** |  | -0.61^#^ | -7.72^#^ | 6.49^#^ | 0.8655 |
| CI: confidence interval; PD: Parkinson’s disease; SHI: statutory health insurance  * Statistical significance (p <0.05)  ^#^ 5-year comparison over the study period 2016–2021 | | | | | |

**Table 2: Rolling 2-year comparisons and overall 5-year^#^ comparison of prevalent PD cases in Germany from the extrapolated cohort**

| **Year** | **Prevalence** | **Prevalence change to previous year** | **Lower CI** | **Upper CI** | **P value** |
| --- | --- | --- | --- | --- | --- |
| *All PD patients* | | | | | |
| **2016** | 575.33 |  | | | |
| **2017** | 568.54 | -6.79 | -19.77 | 6.19 | 0.3054 |
| **2018** | 552.40 | -16.14 | -29.07 | -3.22 | 0.0144* |
| **2019** | 540.32 | -12.08 | -24.92 | 0.76 | 0.0652 |
| **2020** | 519.83 | -20.49 | -33.18 | -7.80 | 0.0015* |
| **2021** | 501.09 | -18.74 | -31.21 | -6.27 | 0.0032* |
| **2016 – 2021*^#^*** |  | -74.24 | -86.93 | -61.55 | <0.0001* |
| *PD patients without a history of dementia* | | | | | |
| **2016** | 368.23 |  | | | |
| **2017** | 364.01 | -4.22 | -14.61 | 6.18 | 0.4265 |
| **2018** | 354.72 | -9.30 | -19.66 | 1.06 | 0.0786 |
| **2019** | 347.36 | -7.35 | -17.66 | 2.95 | 0.1619 |
| **2020** | 341.01 | -6.35 | -16.58 | 3.88 | 0.2240 |
| **2021** | 334.51 | -6.50 | -16.66 | 3.65 | 0.2093 |
| **2016 – 2021*^#^*** |  | -33.72 | -43.99 | -23.46 | <0.0001* |
| *PD patients with history of dementia* | | | | | |
| **2016** | 207.10 |  | | | |
| **2017** | 204.53 | -2.57 | -10.37 | 5.23 | 0.5186 |
| **2018** | 197.69 | -6.85 | -14.60 | 0.91 | 0.0835 |
| **2019** | 192.96 | -4.73 | -12.42 | 2.97 | 0.2284 |
| **2020** | 178.82 | -14.14 | -21.67 | -6.61 | 0.0002* |
| **2021** | 166.58 | -12.24 | -19.50 | -4.97 | 0.0010* |
| **2016 – 2021*^#^*** |  | -40.52 | -48.01 | -33.04 | <0.0001* |
| CI: confidence interval; PD: Parkinson’s disease  * Statistical significance (p <0.05)  ^#^ 5-year comparison over the study period 2016–2021 | | | | | |

**Table 3: Rolling 2-year comparisons and 5-year^#^ comparison of incident PD cases in Germany from the WIG2 database**

| **Year** | **Incidence** | **Incidence change to previous year** | **Lower CI** | **Upper CI** | **P value** |
| --- | --- | --- | --- | --- | --- |
| *All PD patients* | | | | | |
| **2016** | 60.80 |  | | | |
| **2017** | 62.87 | 2.07 | -2.22 | 6.36 | 0.3440 |
| **2018** | 59.62 | -3.25 | -7.54 | 1.04 | 0.1380 |
| **2019** | 62.88 | 3.26 | -1.06 | 7.58 | 0.1389 |
| **2020** | 55.90 | -6.98 | -11.25 | -2.71 | 0.0013* |
| **2021** | 61.95 | 6.05 | 1.79 | 10.30 | 0.0054* |
| **2016 – 2021*^#^*** |  | 1.15 | -3.16 | 5.46 | 0.6014 |
| *PD patients without a history of dementia* | | | | | |
| **2016** | 41.63 |  | | | |
| **2017** | 43.03 | 1.40 | -2.15 | 4.95 | 0.4386 |
| **2018** | 41.62 | -1.41 | -4.98 | 2.16 | 0.4379 |
| **2019** | 44.24 | 2.62 | -1.00 | 6.24 | 0.1555 |
| **2020** | 38.73 | -5.51 | -9.07 | -1.94 | 0.0025* |
| **2021** | 43.62 | 4.89 | 1.33 | 8.45 | 0.0072* |
| **2016 – 2021*^#^*** |  | 1.99 | -1.60 | 5.58 | 0.2774 |
| *PD patients with history of dementia* | | | | | |
| **2016** | 19.17 |  | | | |
| **2017** | 19.84 | 0.67 | -1.74 | 3.08 | 0.5869 |
| **2018** | 18.01 | -1.84 | -4.22 | 0.55 | 0.1316 |
| **2019** | 18.65 | 0.64 | -1.72 | 3.00 | 0.5953 |
| **2020** | 17.17 | -1.48 | -3.82 | 0.87 | 0.2171 |
| **2021** | 18.33 | 1.16 | -1.18 | 3.50 | 0.3301 |
| **2016 – 2021*^#^*** |  | -0.84 | -3.23 | 1.54 | 0.4883 |
| CI: confidence interval; PD: Parkinson’s disease; SHI: statutory health insurance  * Statistical significance (p <0.05)  ^#^ 5-year comparison over the study period 2016–2021 | | | | | |

**Table 4: Rolling 2-year comparisons and overall 5-year^#^ comparison of incident PD cases in Germany from the extrapolated cohort**

| **Year** | **Incidence** | **Incidence change to previous year** | **Lower CI** | **Upper CI** | **P value** |
| --- | --- | --- | --- | --- | --- |
| *All PD patients* | | | | | |
| **2016** | 68.85 |  | | | |
| **2017** | 68.86 | 0.01 | -4.52 | 4.53 | 0.9983 |
| **2018** | 61.92 | -6.94 | -11.38 | -2.51 | 0.0022* |
| **2019** | 63.39 | 1.48 | -2.89 | 5.85 | 0.5081 |
| **2020** | 55.80 | -7.59 | -11.86 | -3.31 | 0.0005* |
| **2021** | 60.30 | 4.50 | 0.27 | 8.72 | 0.0370* |
| **2016 – 2021*^#^*** |  | -8.55 | -12.97 | -4.14 | 0.0001* |
| *PD patients without a history of dementia* | | | | | |
| **2016** | 45.74 |  | | | |
| **2017** | 45.68 | -0.05 | -3.74 | 3.63 | 0.9768 |
| **2018** | 42.54 | -3.15 | -6.79 | 0.50 | 0.0905 |
| **2019** | 44.00 | 1.47 | -2.17 | 5.10 | 0.4291 |
| **2020** | 38.79 | -5.21 | -8.77 | -1.65 | 0.0042* |
| **2021** | 42.41 | 3.62 | 0.09 | 7.16 | 0.0445* |
| **2016 – 2021*^#^*** |  | -3.32 | -6.97 | 0.33 | 0.0745 |
| *PD patients with history of dementia* | | | | | |
| **2016** | 23.12 |  | | | |
| **2017** | 23.18 | 0.06 | -2.56 | 2.68 | 0.9644 |
| **2018** | 19.38 | -3.80 | -6.33 | -1.27 | 0.0033* |
| **2019** | 19.39 | 0.01 | -2.42 | 2.44 | 0.9934 |
| **2020** | 17.01 | -2.38 | -4.74 | -0.02 | 0.0485* |
| **2021** | 17.89 | 0.87 | -1.44 | 3.19 | 0.4599 |
| **2016 – 2021*^#^*** |  | -5.23 | -7.72 | -2.74 | <0.0001* |
| CI: confidence interval; PD: Parkinson’s disease  * Statistical significance (p <0.05)  ^#^ 5-year comparison over the study period 2016–2021 | | | | | |
