# Supplementary material for: Epidemiology of Parkinson's Disease in Germany Between 2016 and 2021 Based on Statutory Health Insurance Claims Data
Source: Brain Behav. 2025 Nov 5;15(11):e71027. doi: 10.1002/brb3.71027 (PMC12589807; doi:10.1002/brb3.71027)
Supplement: Supplementary file 2 — Supplementary Material: brb371027‐sup‐0002‐appendix.docx [file BRB3-15-e71027-s001.docx]

**Supplementary Appendix**

**Parkinson’s Disease / Parkinson’s Disease with motor fluctuation / Dementia**

The following diseases were identified as having either one main inpatient diagnosis and/or two confirmed outpatient respectively secondary inpatient diagnoses as shown in Table 1.

Table 1: Identification patterns for diseases based on ICD-10-GM codes

| ICD-10 | Definition |
| --- | --- |
| **Parkinson’s Disease** | |
| G20 | Primary Parkinson's syndrome |
| G200 | Primary Parkinson's syndrome with absent or minor impairment |
| G2000 | Primary Parkinson's syndrome with absent or minor impairment: Without motor fluctuation |
| G2001 | Primary Parkinson's syndrome with absent or minor impairment: With motor fluctuation |
| G201 | Primary Parkinson's syndrome with moderate to severe impairment |
| G2010 | Primary Parkinson's syndrome with moderate to severe impairment: Without motor fluctuation |
| G2011 | Primary Parkinson's syndrome with moderate to severe impairment: With motor fluctuation |
| G202 | Primary Parkinson's syndrome with most severe impairment |
| G2020 | Primary Parkinson's syndrome with most severe impairment: Without motor fluctuation |
| G2021 | Primary Parkinson's syndrome with most severe impairment: With motor fluctuation |
| G209 | Primary Parkinson's syndrome, unspecified |
| G2090 | Primary Parkinson's syndrome, unspecified: Without motor fluctuation |
| G2091 | Primary Parkinson's syndrome, unspecified: With motor fluctuation |
| **Parkinson’s Disease with motor fluctuation** | |
| G2001 | Primary Parkinson's syndrome with absent or minor impairment: With motor fluctuation |
| G2011 | Primary Parkinson's syndrome with moderate to severe impairment: With motor fluctuation |
| G2021 | Primary Parkinson's syndrome with most severe impairment: With motor fluctuation |
| G2091 | Primary Parkinson's syndrome, unspecified: With motor fluctuation |
| **Dementia** | |
| F00 | Dementia in Alzheimer's disease |
| F000 | Dementia in Alzheimer's disease, with early onset (type 2) |
| F001 | Dementia in Alzheimer's disease, with late onset (type 1) |
| F002 | Dementia in Alzheimer's disease, atypical or mixed form |
| F009 | Dementia in Alzheimer's disease, unspecified |
| F01 | Vascular dementia |
| F010 | Vascular dementia with acute onset |
| F011 | Multi-infarct dementia |
| F012 | Subcortical vascular dementia |
| F013 | Mixed cortical and subcortical vascular dementia |
| F018 | Other vascular dementia |
| F019 | Vascular dementia, unspecified |
| F02 | Dementia associated with diseases classified elsewhere |
| F020 | Dementia in Pick's disease |
| F021 | Dementia in Creutzfeldt-Jakob disease |
| F022 | Dementia in Huntington's disease |
| F023 | Dementia in primary Parkinson's syndrome |
| F024 | Dementia in HIV [human immunodeficiency virus disease]. |
| F028 | Dementia associated with diseases classified elsewhere |
| F03 | Unspecified dementia |
| F051 | Delirium in dementia |
| G30 | Alzheimer's disease |
| G300 | Alzheimer's disease with early onset |
| G301 | Alzheimer's disease with late onset |
| G308 | Other Alzheimer's disease |
| G309 | Alzheimer's disease, unspecified |
| G311 | Senile degeneration of the brain, not elsewhere classified |
